# Knowledge and attitude towards Ebola and Marburg virus diseases in Uganda using quantitative and participatory epidemiology techniques

**DOI:** 10.1371/journal.pntd.0005907

**Published:** 2017-09-11

**Authors:** Luke Nyakarahuka, Eystein Skjerve, Daisy Nabadda, Doreen Chilolo Sitali, Chisoni Mumba, Frank N. Mwiine, Julius J. Lutwama, Stephen Balinandi, Trevor Shoemaker, Clovice Kankya

**Affiliations:** 1 Department of Food Safety and Infection Biology, Norwegian University of Life Sciences, Oslo, Norway; 2 College of Veterinary Medicine, Animal Resources and Biosecurity, Department of Biosecurity, Ecosystems and Veterinary Public Health, Makerere University, Kampala, Uganda; 3 Department of Arbovirology, Emerging and Re-emerging diseases, Uganda Virus Research Institute, Entebbe, Uganda; 4 University of Zambia, School of Veterinary Medicine, Department of Disease Control, Lusaka, Zambia; 5 College of Veterinary Medicine, Animal Resources and Biosecurity, Department of Biomolecular Resources and Biolab Sciences, Makerere University, Kampala, Uganda; 6 Viral Special Pathogens Branch, United States Centers for Disease Control and Prevention(CDC), Viral Hemorrhagic Fever Laboratory, Entebbe, Uganda; Armed Forces Health Surveillance Center, UNITED STATES

## Abstract

**Background:**

Uganda has reported five (5) Ebola virus disease outbreaks and three (3) Marburg virus disease outbreaks from 2000 to 2016. Peoples’ knowledge and attitude towards Ebola and Marburg virus disease impact on control and prevention measures especially during outbreaks. We describe knowledge and attitude towards Ebola and Marburg virus outbreaks in two affected communities in Uganda to inform future outbreak responses and help in the design of health education and communication messages.

**Methods:**

The study was a community survey done in Luweero, Ibanda and Kamwenge districts that have experienced outbreaks of Ebola and Marburg virus diseases. Quantitative data were collected using a structured questionnaire and triangulated with qualitative participatory epidemiology techniques to gain a communities’ knowledge and attitude towards Ebola and Marburg virus disease.

**Results:**

Out of 740 respondents, 48.5% (359/740) were categorized as being knowledgeable about Ebola and Marburg virus diseases, whereas 60.5% (448/740) were having a positive attitude towards control and prevention of Ebola and Marburg virus diseases. The mean knowledge and attitude percentage scores were 54.3 (SD = 23.5, 95%CI = 52.6–56.0) and 69.9 (SD = 16.9, 95%CI = 68.9–71.1) respectively. People educated beyond primary school were more likely to be knowledgeable about Ebola and Marburg virus disease than those who did not attain any formal education (OR = 3.6, 95%CI = 2.1–6.1). Qualitative data revealed that communities describe Ebola and Marburg virus diseases as very severe diseases with no cure and they believe the diseases spread so fast. Respondents reported fear and stigma suffered by survivors, their families and the broader community due to these diseases.

**Conclusion:**

Communities in Uganda affected by filovirus outbreaks have moderate knowledge about these diseases and have a positive attitude towards practices to prevent and control Ebola and Marburg viral diseases. The public health sector should enhance this community knowledge gap to empower them more by supplying educational materials for epidemic preparedness in future using appropriate communication channels as proposed by the communities.

## Author summary

Ebola Virus Disease (EVD) and Marburg Virus Disease (MVD) are caused by a family of viruses known as Filoviruses. When they occur, they cause high lethality among infected people, which causes panic to the population, as well as interfering with the health care delivery system, especially in developing countries. Usually, at the beginning of these infections, the affected communities tend to think that witchcraft or some bad luck has befallen their community. Because of limited knowledge about these diseases, it becomes hard for local authorities to institute control and prevention measures. We administered a questionnaire to individual participants and held focus group discussions to help us tease out the communities’ understanding of these diseases. We found that people have moderate knowledge about EVD and MVD, and a positive attitude towards the prevention and control measures instituted by health authorities in Uganda. However, they still reported stigma subjected to the survivors of these diseases and affected families at large. EVD still causes much fear which drives some of the irrational actions by communities during outbreaks. Communities highlighted early sensitization as a means of controlling outbreaks. We recommend the findings in this paper to public health authorities in epidemic-prone countries like Uganda to aid in control and epidemic preparedness of filovirus outbreaks.

## Introduction

Ebola and Marburg virus diseases are viral hemorrhagic fevers (VHFs) known to cause high morbidity and mortality and pose a serious threat to human and animal populations in endemic countries. These classical VHFs are caused by filoviruses that belong to the family *Filoviridae*. A total of 28,646 people were reported to be infected with Ebola virus in the recent outbreak in West Africa in 2014, out of which 11,323 died [1]. Apart from causing morbidity and mortality, outbreaks of VHFs cause panic among the public, interfere with global travel and have a devastating socio-economic impact [2, 3]. Uganda has reported five (5) Ebola virus disease (EVD) outbreaks and three (3) Marburg virus disease (MVD) outbreaks since 2000. The first EVD outbreak in the year 2000 remains the largest EVD outbreak ever recorded in Uganda, during which 425 cases and 224 deaths (CFR 53%) were reported [4]. Since then, four (4) additional EVD outbreaks have occurred; one in Bundibugyo district in 2007 caused by *Bundibugyo ebolavirus* (116 cases, 39 deaths) [5]. Other outbreaks happened in Luweero district in 2011 (one case, one death) [6], in Kibaale district in 2012 (11 confirmed cases, four deaths) and Luweero district again in 2012 (6 cases and three deaths) [7].

Three (3) MVD outbreaks have been reported in Uganda. The first recorded outbreak was in 2007, where three (3) cases and one (1) death were reported in a community associated with mining activities in the districts of Kamwenge and Ibanda, Western Uganda [8]. In 2012, MVD was responsible for 26 cases with 15 deaths affecting multiple districts [9]. In 2014, Uganda reported only one case diagnosed with Marburg virus in Kampala (Uganda’s capital city) [10]. These outbreaks are believed to occur because of close interaction of people and animals such as non-human primates, bats, and livestock. Previous studies in Uganda have demonstrated bats of species *Rousettus aegyptiacus* to be the known reservoir for Marburg virus [11–13]. In Uganda, this bat species has been found in the Kitaka mine in Ibanda district as well as in Maramagambo “python cave” in the neighboring Rubirizi district, as well as other sites in the surrounding areas. Two tourists visiting python caves were infected with Marburg virus in 2008 with one fatality [14–16].

These outbreaks cause loss of human life, associated morbidities and induce stress on the socio-cultural and health care systems as efforts to respond to these outbreaks require many resources such as funds, laboratory testing, and personnel. Usually, when these outbreaks occur, health care workers run away from health facilities leaving patients with no health care and support due to lack of protective equipment, fear of contracting the disease and stigmatization from their families [17].

Research done in West Africa by Iliyasu *et al*. [18] showed suboptimal knowledge, attitudes and practices towards EVD, and associated myths and misconceptions which negatively impacted the response mechanisms. The stigma associated with communicable diseases like EVD interfere with control and prevention of these diseases as observed by Davtyan *et al*. [19]. Many people are reluctant to associate themselves with EVD survivors. This was the situation in the 2014 West African EVD outbreak [20, 21], also reported by deVries *et al*. 2016 in Luweero district of Uganda [22]. However, as an outbreak progresses, people tend to modify their behavior. For example, an outbreak of EVD that happened in Uganda in Masindi District 2000, the case fatality rate was high at the beginning of the outbreak (76%) but decreased to 20% at the end of the epidemic as people started modifying their behavior towards the epidemic. [23].

In Uganda, EVD survivors reported fear, ostracism, and stigmatization from their community [24]. There is always an over-reaction in communities characterized by anger, fear and the communities tend to run away from hospitals searching for spiritual healing as they associate EVD or MVD with witchcraft also locally known as “*amayembe*”[22]. These actions are counterproductive towards efforts to control the spread of VHFs.

For a better response to future EVD and MVD outbreaks in Uganda, there is a need to better understand communities’ knowledge and attitudes towards these VHFs. Therefore, our main objective was to describe knowledge and attitudes in two communities affected by outbreaks of Ebola and Marburg viral diseases in Uganda. This information may be critical in designing health education, information, and communication materials in future outbreaks, leading to better control of future epidemics.

## Methods and materials

### Study site, study population, and sampling strategy

**Fig 1:**
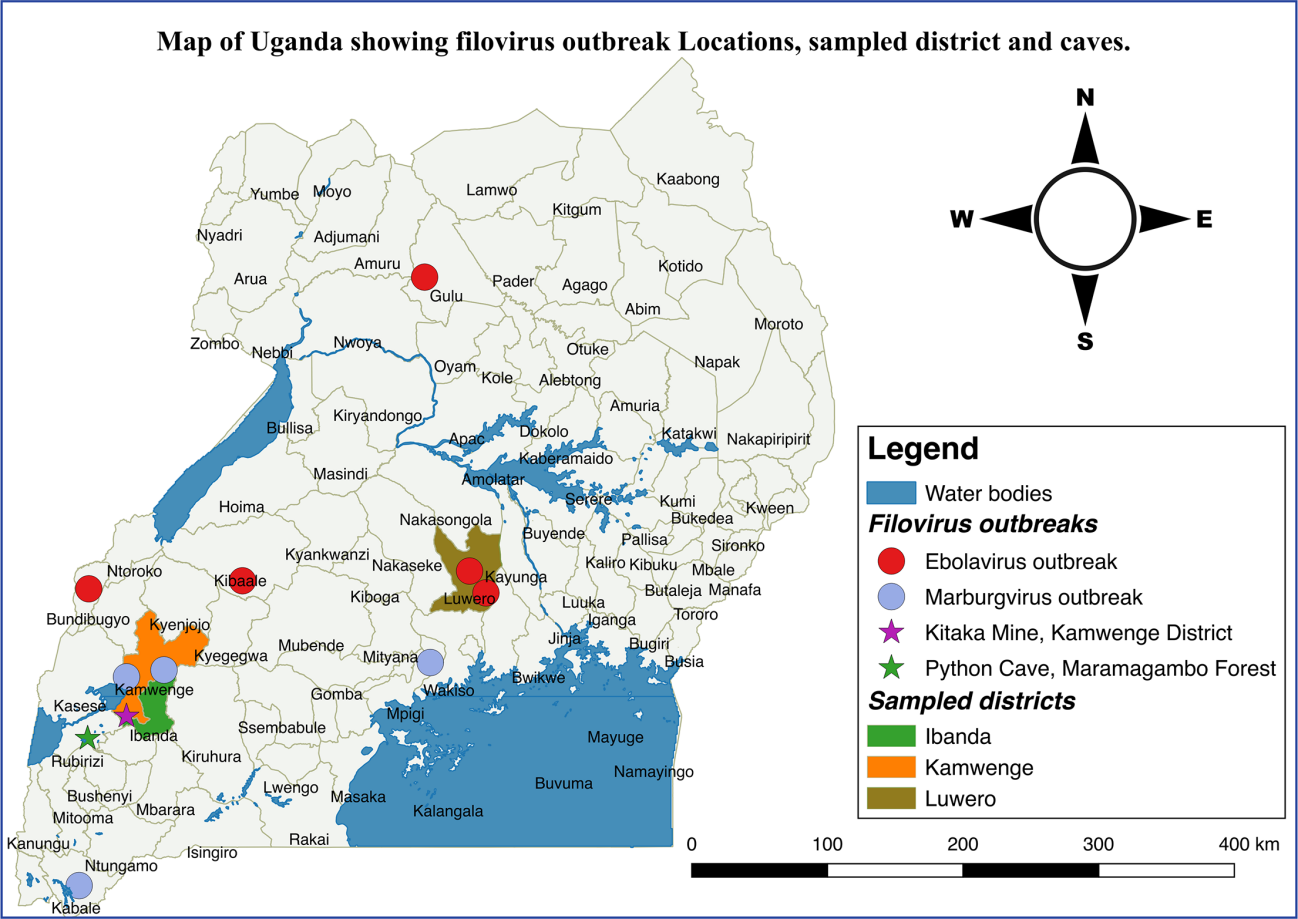
Map of Uganda showing Ebola and Marburg disease outbreaks and study districts (map developed in QGIS desktop software, the base layers from Uganda bureau of statistics-http://www.ubos.org/statistical-activities/gis/).

The study was undertaken in two different locations in Uganda in the months of January and February 2015, as part of a larger study intended to assess the seroprevalence of MVD and EVD in high-risk areas in Uganda (Fig 1). First, we focused on communities in Western Uganda in the districts of Kamwenge and Ibanda that were affected by MVD outbreaks twice, the first one in 2007 and another one in 2012. The second study site was in Luweero district, Central Uganda that has been affected by EVD outbreaks twice, one in 2011 and another in 2012 [6, 7]. The main economic activity in the two sites is agriculture, mainly crop farming and livestock keeping.

We estimated the necessary sample size using StatCalc, an application in the EpiInfo software, which gave us 768 study participants, 384 in each study area, based on an expected proportion of the population that have knowledge about MVD and EVD at 50% and the desired precision of 5%. However, only 740 completed the questionnaires representing a response rate of 96.4%. We studied the population in Ibanda, Kamwenge and Luweero districts, purposively sampling villages that were affected by Ebola and Marburg virus diseases outbreaks. A multisector team of members from the Uganda Virus Research Institute (UVRI), Ministry of Health Uganda, Makerere University Kampala Uganda and US Centers for Disease Control and Prevention Uganda working with district health teams visited affected villages to recruit study participants. Through working with local community leaders and health workers, a snowball approach was used to recruit participants. Participants for the questionnaire were chosen using convenient sampling, and both communities were asked the same questions.

### Quantitative data collection

Research assistants were trained to use a structured questionnaire to collect data (S1 Questionnaire). Participants were asked to give a written consent after the objectives of the study were explained to them before the questionnaire could be administered. The questionnaire was pre-tested in Wakiso district that was not part of the survey to ensure that validity and clarity of the questions, and minor editing was done to get a final questionnaire. The questionnaire consisted of three sections, socio-demographic characteristics, practices that predispose people to EVD and MVD, knowledge and attitude questions. Closed-ended questions were used to assess peoples’ knowledge and attitudes on transmission and risk factors, prevention and control, causation, signs and symptoms and treatment of MVD and EVD. Questionnaires were administered in the local language to one person per household that lived in sub-counties that had reports of EVD and MVD outbreaks but not to survivors or their family members as these were targeted for qualitative data collection.

### Attitude and knowledge scoring

Knowledge and attitude questions that were answered correctly were scored one (1) while those that were answered wrongly were scored zero (0). All questions were given equal weight, and missing responses were not scored, whereas “do not know” responses were scored zero (0). The knowledge and attitude score for each study participant were used to compute the percentage scores out of a total score of 34 and 20 respectively. The validity of the knowledge and attitude questions was confirmed by an adequate Cronbach's alpha internal consistency measured at 0.90.

### Quantitative statistical analysis

**Fig 2:**
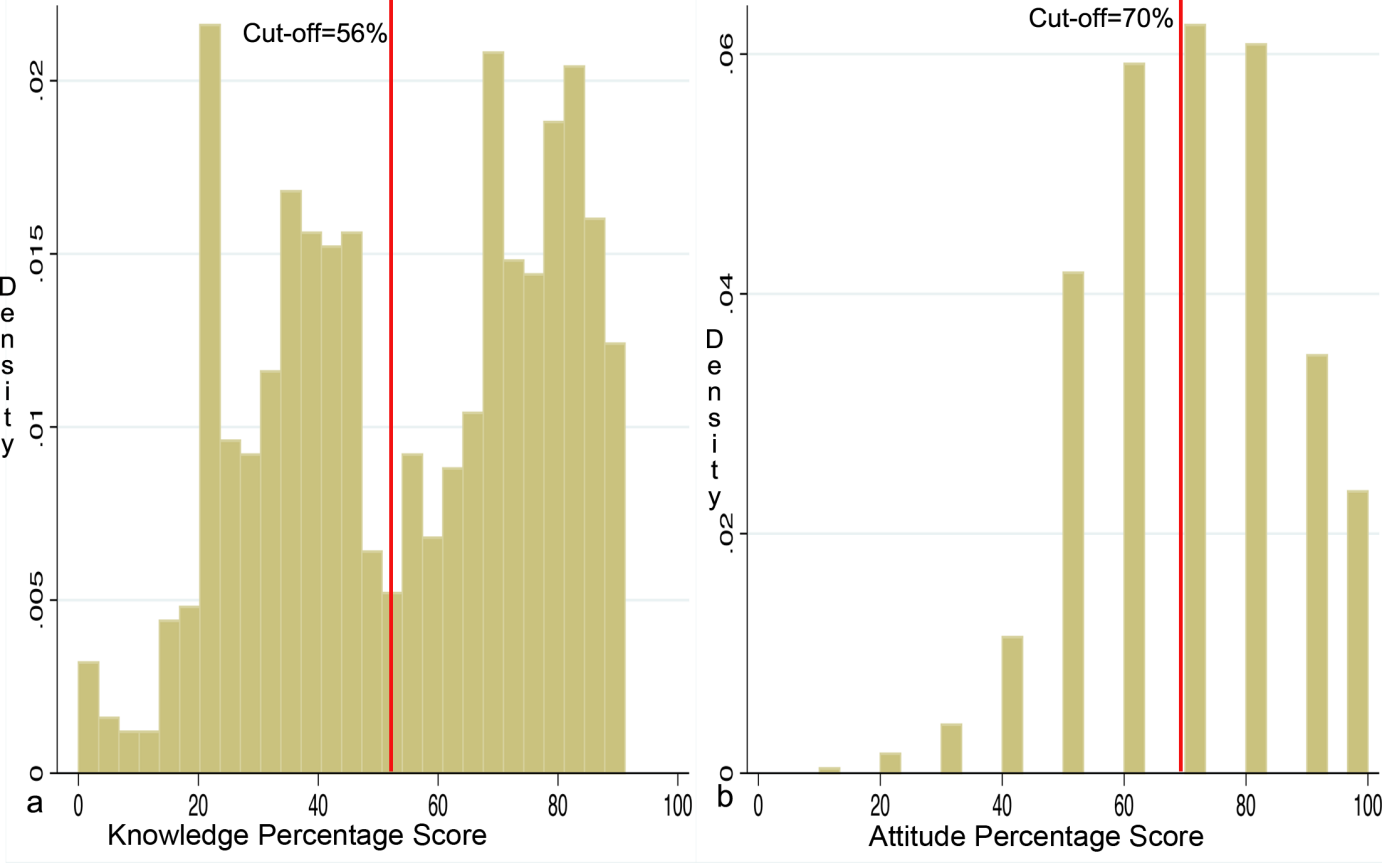
a: Distribution of percentage knowledge scores; the red line shows cut-off set at a median score of 56%. b: Distribution of percentage attitude scores; the red line shows cut-off set at a median score of 70%.

Data were entered into EpiInfo software, assessed for normality and univariate analysis was done and later exported to Stata (Stata/ SE for Windows, StataCorp, College Station, TX) for further analysis. Results are presented in tables and narratives. A cut-off point was set based on percentage knowledge and attitude distribution, and median scores as was described in other studies [18, 25]. For knowledge score, the median percentage score was 56%; with a bimodal curve distribution of the scores, hence those below a 56% score were categorized as having poor knowledge and those with 56% and above score as having good knowledge (Fig 2A). Further, attitudes were classified as being negative if the percentage score was below the median score of 70% and positive if the median score was 70% and above (Fig 2B). The relationship between good or poor knowledge and attitude was explored using a univariable logistic regression. Predictors of good versus poor knowledge with a p-value of 0.2 and below were included in a multivariable logistic regression model to determine the predictors of good knowledge towards EVD and MVD. The model was constructed using a backward selection procedure using the likelihood ratio test (LRT) with a p = 0.05 for keeping a variable in the model. Model evaluation was done using the Hosmer-Lemeshow test of goodness of fit and the area under the receiver operating curve (ROC).

### Participatory epidemiology data collection techniques

Qualitative participatory appraisal techniques, also known as Participatory Epidemiology (PE) were used to triangulate the findings of quantitative data collection. Five (5) focus group discussions (FGDs) involving 50 participants were conducted. FGDs were held within rural communities that were affected by outbreaks, drawn mainly from survivors of EVD and MVD and their family members, community and opinion leaders, as well as other members of the community who were 18 years and above. The discussions involved both male and female respondents since gender disaggregation was not the focus of this study.

An introduction explaining the purpose of the exercise was carried out with the informants before conducting the interview. Semi-structured interview guides were translated into the community’s local language (Luganda and Runyankore) by trained research assistants and were used to gain an understanding of the local perception of Ebola and Marburg virus diseases (S1 FGD guide). To get a clear knowledge of the community’s knowledge and attitude towards EVD and MVD, we subjected the information generated from FGD guide to three PE tools, which included simple ranking, proportional piling, and pairwise ranking. Simple ranking techniques helped us to understand what the community considered as the most important depending on the topic being discussed. For example, the FGD participants were asked to list what they believed to be the clinical symptoms of EVD and MVD and later requested to rank them from the most important to the least important according to their opinion (S1 Fig). Proportional piling was used to study what the community thinks are modes of transmission of filoviruses. Here, participants were given 100 grains of beans and were required to distribute them according to the importance of the factor being discussed. Informants did not count the beans; rather they simply piled the beans judging by the importance of the mode of transmission in spreading filoviruses (S2 Fig). The pairwise ranking technique was used to understand the communities’ ideas on the source, cause or the triggers of EVD and MVD outbreaks. Pairwise ranking technique compared each proposed source or cause of the outbreaks with each other systematically and then ranking was done to see what the community considers as the most important cause of outbreaks in their communities (S3 Fig). Agreement within FGDs participants was reached by consensus.

Discussions from FGDs were audiotaped with permission from informants and transcribed verbatim. Data generated through FGDs were analyzed using conventional content analysis as reported by Hsieh and Shannon [26] where qualitative data was merged into codes, categories and themes. Text data were read several times to get a deeper understanding of the emerging codes and categories. Categories were later grouped into topics such as participants understanding of Ebola and Marburg virus diseases, modes of transmission, clinical symptoms, the impact of outbreaks, communication, prevention, and control.

### Ethics statement

Approval to conduct this study was obtained from Uganda Virus Research Institute Research and Ethics Committee and Uganda National Council of Science and Technology (UNCST approval NO: HS 1538). Participants gave signed written consent to participate in this study. For participants under the age of 18 years, informed consent was provided by their parents or their guardians on their behalf.

## Results

### Socio-demographic characteristics of questionnaire survey participants

Of the 740 participants who completed the questionnaire, 60% were from Western Uganda in communities affected by MVD in Ibanda and Kamwenge districts and 40% were from Central Uganda in EVD affected communities of Luweero district. Overall, 54.2% were males, 16.8% had never attended any formal education, and the majority (62.7%) occupation was farming. The median age was 33 years (range 3–82 years), and 85.2% were above 20 years. These statistics are close to those of Uganda population census 2014 from these districts.

### Knowledge on Ebola and Marburg viral diseases and their modes of transmission

Table 1 highlights some of the responses from participants on questions assessing knowledge about Ebola and Marburg virus diseases and their modes of transmission. Almost all (96.2%) had heard about EVD and MVD, 43.5% reported to know how to identify a suspect case of EVD and MVD, the most known clinical symptom for EVD and MVD was bleeding at 54.3%, and 28.2% reported to know a survivor of EVD and MVD.

**Table 1:** Knowledge on Ebola and Marburg viral diseases and their modes of transmission.

**Variable**	**n/N**	**Percent (%)**	**95% Confidence Limits**
**Have heard about Ebola & Marburg virus diseases**			
Yes	712/740	96.2%	94.5%-97.4%
No	28/740	3.8%	2.6%-5.5%
**Source of information about Ebola & Marburg virus diseases**			
Health worker	113/724	15.6%	13.1%-18.4%
Radio	614/726	84.6%	81.6%-87.1%
Community leaders	146/724	20.2%	17.3%-23.3%
Other sources of communication	64/720	8.9%	6.9%-11.3%
**Know symptoms of Ebola and Marburg virus disease suspects**			
No	392/740	53.0%	49.3%-56.6%
Not Sure	26/740	3.5%	2.3%-5.2%
Yes	322/740	43.5%	39.9%-47.2%
**Known symptoms of Ebola and Marburg virus disease**			
Bleeding	277/510	54.3%	49.8%-58.7%
Fever	106/508	20.9%	17.4%-24.7%
Vomiting	100/509	19.7%	16.3%-23.4%
Diarrhea	87/506	17.2%	14.1%-20.8%
Other signs	52/501	10.4%	7.9%-13.5%
**Know whom to contact for suspect case of Ebola and Marburg virus diseases**			
Yes	50/740	6.8%	5.1%-8.9%
No	690/740	93.2%	91.13%- 94.9%
**Know a survivor of Ebola or Marburg virus diseases**			
Yes	209/740	28.2%	25.1%- 31.7%
No	531/740	71.8%	68.3%-75.0%
**Know how Ebola or Marburg virus diseases are transmitted**			
No	327/739	44.3%	40.6%-47.9%
Not sure	35/739	4.7%	3.4%-06.6%
Yes	377/739	51.0%	47.4%-54.7%
**Known modes of transmission of Ebola and Marburg virus disease**			
Body contact with Ebola infected person	289/533	54.2%	49.9%-58.5%
Through air	90/533	16.9%	13.7%-20.4%
Through needle pricks	71/532	13.4%	10.6%-16.6%
Contact with animals	162/534	30.3%	26.5%- 34.5%
From a person who died of EVD or MVD	122/532	22.9%	19.5%-26.8%
Contact with body fluids of sick person	134/533	25.1%	21.6%-29.1%
Biting mosquitoes	60/530	11.3%	8.8%-14.4%
Other means of transmission	40/524	7.6%	5.6%-10.3%
**Think one can get infection from asymptomatic Ebola or Marburg virus disease suspects**			
No	167/530	31.5%	27.6%-35.7%
Not sure	19/530	3.6%	2.2%-5.6%
Yes	344/530	64.9%	60.7%-68.9%
**Think one can acquire Ebola or Marburg virus disease from contact with bush meat**			
No	203/543	37.4%	33.3%-41.6%
Not sure	42/543	7.7%	5.7%-10.4%
Yes	298/543	54.9%	50.6%-59.1%
**Think one can get Ebola or Marburg virus disease from eating fruits eaten on by bats**			
No	201/541	37.2%	33.1%-41.4%
Not sure	40/541	7.4%	5.4%-10.0%
Yes	300/541	55.5%	51.2%-59.7%
**Think one gets infected from sexual fluids of a person who recovered from infection**			
No	202/540	37.4%	33.3%-41.2%
Not sure	63/540	11.7%	9.1%-14.8%
Yes	275/540	50.9%	46.6%-55.3%
**Think one gets infected from breast milk of an infected person or survivor**			
No	207/539	38.4%	34.3%-42.7%
Not sure	45/539	8.4%	6.2%-11.1%
Yes	287/539	53.3%	48.9%-57.5%
**Shaking hands/physical contact with a person infected with Ebola or Marburg viruses**			
No	167/538	31.0%	27.2%-35.5%
Not sure	20/538	3.7%	2.4% -5.8%
Yes	351/538	65.2%	61.0%-69.2%

On the mode of transmission, 51% knew how EVD and MVD are transmitted. A total of 54.2% knew that EVD or MVD could be transmitted through body contact with an infected person, while 11.3% thought that EVD/MVD could be transmitted through biting mosquitoes, 16.7% thought that EVD/MVD are airborne, 50.9% mentioned that it could be transmitted through semen or sexual contact and 53.3% knew that EVD/MVD could be transmitted through breast milk of an infected person.

### Knowledge on control and prevention of Ebola and Marburg virus diseases

A total of 62.8% (465/740) reported to know how EVD and MVD could be controlled and prevented, 52.8% (362/686) said by avoiding sick people, 39.4% (270/686) by avoiding contact with animals and 11.1% (76/686) by vaccination. Only 4.5% (24/531) knew that EVD and MVD are caused by a virus, 58.7% thought that it is caused by wildlife such as primates and monkeys whereas only 1.1% attributed it to witchcraft as shown in Table 2.

**Table 2:** Knowledge on control and prevention of Ebola and Marburg virus disease.

Variable	n/N	Percent (%)	95% Confidence Limits
**Reported to know control and prevention measures**			
No	218/740	29.5%	26.2%-32.9%
Not sure	57/740	7.7%	5.9%-9.9%
Yes	465/740	62.8%	59.2%-66.3%
**Known control and prevention measures**			
Vaccination	76/686	11.1%	8.9%-13.7%
Avoiding contact with animals	270/686	39.4%	35.7%-43.1%
Traditional medicine	22/685	3.2%	2.1%-4.9%
Avoiding sick people	362/686	52.8%	48.0%-56.6%
Other means	90/686	13.1%	10.7%-15.9%
**Know the cause of Ebola and Marburg viruses**			
No	323/727	44.4%	40.8%-48.1%
Not sure	52/727	7.2%	5.4%-9.3%
Yes	352/727	48.4%	44.7%-52.1%
**Known causes of Ebola and Marburg viruses**			
Virus	24/531	4.5%	3.0%-6.8%
Bats, monkey or other wild animals	312/532	58.7%	54.3%-62.9%
God or other higher power	9/531	1.7%	0.8%-3.3%
Witchcraft	6/531	1.1%	0.5%-2.6%
Evil-doing	4/531	0.8%	0.2%-2.1%
Curse	4/531	0.8%	0.2%-2.1%
**Prevention by avoiding contact with body fluids**			
No	157/596	26.3%	22.9%-30.1%
Not sure	019/596	3.2%	2.0%-5.0%
Yes	420/596	70.5%	66.6%-74.1%
**Prevention by avoiding funerals**			
No	180/590	30.5%	26.9%-34.4%
Not sure	33/590	5.6%	3.9%-7.9%
Yes	377/590	63.9%	59.9%-67.8%
**Prevention by reporting suspects to hospital**			
No	162/598	27.1%	23.6%-30.9%
Not sure	26/590	4.4%	2.9%-6.4%
Yes	410/590	68.6%	64.7%-72.3%

### Attitudes towards Ebola and Marburg viral disease

Regarding attitude, 87.3% (646/740) of participants believed that EVD and MVD actually exist, 52.7% (386/733) would not relate with a survivor of Ebola or Marburg virus disease. The fear of contracting the disease was the main reason for not associating with EVD, or MVD survivors representing 59.6% (334/560), 24.7% (182/736) would not welcome a survivor back into the community as shown in Table 3.

**Table 3:** Attitudes towards Ebola and Marburg viral disease.

Variable	Frequency (n/N)	Percent (%)	95% Confidence Interval
**Believe that Ebola and Marburg viral diseases really exists**			
No	48/740	6.5%	4.9%-8.6%
Not sure	46/740	6.2%	4.6%-8.3%
Yes	646/740	87.3%	84.3–89.6%%
**Would relate with survivor of Marburg and Ebola viral disease**			
No	386/733	52.7%	49.0%-56.3%
Not sure	26/733	3.6%	2.4%-5.2%
Yes	321/733	43.8%	40.2%-47.5%
**Why they would not relate with survivor of EVD/MVD**			
Fear of contracting the disease	334/560	59.6%	55.4%-63.7%
Fear of stigma from community	18/554	3.3%	2.0%-5.2%
Other reasons	6/551	1.1%	0.4%-2.5%
**How Ebola and Marburg viral diseases should be treated**			
Traditional African medicine	3/706	0.4%	0.1%-1.3%
Spiritual healing	8/710	1.1%	0.5%-2.3%
Modern Western medicine	652/711	91.7%	89.4%-93.6%
Herbal medicine	5/708	0.7%	0.3%-1.7%
Other modes of treatment	17/704	2.4%	1.5%-3.9%
**Think are at risk of infection with Ebola or Marburg virus diseases**			
No	159/739	21.5%	18.6%-24.7%
Not sure	67/739	9.1%	7.1%-11.4%
Yes	513/739	69.4%	65.9%-72.7%
**Would buy from a shopkeeper who is a survivor**			
No	298/740	40.3%	36.7%-43.9%
Not sure	29/740	3.9%	2.7%-5.7%
Yes	413/740	55.8%	52.1%-59.4%
**Would keep information secret if family member is suspected to be infected with EVD or MVD**			
No	465/722	64.4%	60.8%-67.9%
Not sure	25/722	03.5%	2.3%-5.5%
Yes	232/722	32.1%	28.8%-35.7%
**Would welcome back a survivor of Ebola or Marburg virus disease into the community**			
No	182/736	24.7%	21.7%-28.0%
Not sure	26/736	3.5%	2.4%-5.2%
Yes	528/736	71.7%	68.3%-74.9%

### Overall knowledge and attitude towards Ebola and Marburg Virus diseases

Out of 740 respondents, 48.5% (359/740) were categorized as being knowledgeable about Ebola and Marburg virus diseases whereas 60.5% (448/740) as having a positive attitude towards control and prevention of Ebola and Marburg viral diseases. The mean knowledge and attitude percentage scores were 54.3 (95%CI = 52.6–56.0) and 69.9 (95% CI = 68.9–71.1) respectively.

Table 4 shows results from the logistic regression model for the predictors of knowledge about EVD and MVD, identified as being male, attaining secondary and post-secondary levels of education. The Hosmer-Lemeshow test for goodness of fit shows that the model fits very well the data (P-value = 0.93), and area under the ROC curve = 0.71(S4 Fig). Results of the regression model using uncategorized knowledge percentage scores were the same as the logistic regression model(S3 Table).

**Table 4:** Logistic regression model for predictors of knowledge about EVD and MVD in Uganda (Hosmer-Lemeshow χ^2^ = 1.31; p-value = 0.93, area under the ROC curve = 0.7).

Variable	Poor Knowledge (%)	Good Knowledge (%)	Total	Crude OR(95%CI)	Adjusted^a^ OR (95% CI)
**Gender**					
Female	216(63.9%)	122(36.1%)	338(45.7%)	Ref	
Male	165(41.0%)	237(58.9%)	402(54.3%)	2.5(1.9–3.4)*	1.9(1.4–2.6)*
**Education Level**					
Never attained formal Education	87(70.2%)	37(29.8%)	124(16.8%)	Ref	
Primary level of education	223(54.7%)	185(45.3%)	408(55.1%)	1.9(1.3–3.0)*	1.5(0.9–2.4)
Secondary level of education	67(35.5%)	122(65.5%)	189(25.5%)	4.3(2.6–6.9)*	3.8(2.3–6.3)*
Tertiary level of education	4(21.1%)	15(78.9%)	19(2.6%)	8.8(2.7–28.4)*	8.4(2.5–27.5)*
**Occupation**					
Non-miners	329(57.1%)	247(42.9)	577(77.8%)	Ref	
Miners	52(31.7%)	112(68.3)	164(22.2%)	2.9(1.9–4.1)*	2.6(1.7–3.8)*

*statistically significant

^a^adjusted for all the variables in the model

### Participatory epidemiology results

#### Peoples’ Beliefs about Ebola and Marburg virus diseases

Participants were asked questions regarding knowledge and attitude towards Ebola and Marburg virus diseases, and their discussions are summarized in S1 Table. People believed that Ebola and Marburg viral diseases kill instantly, cause chaos, and are more severe than HIV. There is much fear when the word “Ebola” is mentioned as it is considered a terrible disease.

“*When I hear Ebola*, *I lose strength because it kills instantly*,” said one of the participants in FGD 2.

“*When you get Ebola*, *your life ends there*,” retorted another participant in FGD 1. The details of themes, categories, and quotes are presented in supporting information file(S1 Table)

#### Knowledge on cause and mode of transmission of Ebola and Marburg viral diseases

Almost all the participants agreed that EVD and MVD spread very fast and are highly infectious and contagious diseases. They appreciated the need not to conduct and participate in any funeral rites whenever loved ones die. However, they found this position very hard to accept.

Communities identified non-human primates such as monkeys, chimpanzees and other wildlife such as bats as sources of Ebola and Marburg viral outbreaks as shown by pairwise ranking in S2 Table. However, some people believe that EVD and MVD are transmitted through the air (airborne), poor hygiene, and some think foreign doctors can spread it by malice.

Table 5 shows the results of simple ranking procedure of what the communities believe are the clinical signs of Ebola and Marburg virus disease. Almost 50% think EVD is transmitted by contact with infected person. Top-ranked signs include bleeding from body orifices and other related hemorrhagic signs, diarrhea and vomiting respectively. However, survivors believed that EVD and MVD usually start like malaria with fever.

**Table 5:** Results of simple ranking and proportional piling as listed and ranked by five FGD participants.

Simple Ranking of Clinical Signs of Ebola and Marburg viral diseases		Impact of Ebola and Marburg viral diseases		Preferred means of communication during outbreaks		Proportional piling of modes of transmission	
Clinical signs listed by Participants	Rank score	Impact listed by participants	Rank Score	Means of communication listed by Participants	Rank Score	Mode of transmission listed by participants	Percentage (%)
Bleeding from body orifices	1.0	Fear of Death	1.0	Community Public Radios	1	Handshaking	49%
Diarrhea	3.0	Stigma	2.5	FM Local Radio	2	Being near an Ebola patient	22%
Vomiting	3.0	Reduced income	3.3	Village cooperative societies	3	Attending funerals	14%
Body weakness	4.3	Could not participate in Funeral rites	4.0	Local leaders	4	Taking care of the sick patients	8%
Headache	5.0	Not knowing the cause	4.0	Village meetings	5	Contact with body fluids	4%
Red eyes	5.0	No partying	4.0	TV	6	Sex with Ebola patients/Survivor	3%
Fever	5.3	Death of people	5.0	Newspapers	7		
Anorexia	6.7	Orphans	6.0	Posters	8		
Sweating	6.3			Facebook	9		
Abdominal pain	6.7						
Sudden death	7.3						

“*I have never seen such a deadly disease since my daughter started falling sick with a simple fever and we all thought it was malaria*,*”* said one of the participants who took care of an EVD patient in FGD 3.

#### Knowledge on control and prevention

Community sensitization was a major proposal fronted by the community as a way of controlling Ebola and Marburg viral diseases. They emphasize repetitive sensitization for the population to be aware of the diseases. They also emphasize safe burial of their loved ones to increase compliance. Other means of prevention and control suggested by the community include quarantine, treatment, recruitment of health workers who are qualified to handle EVD and MVD. They also proposed the elimination of bats and rats and other wildlife that they believed to be causes of Marburg and Ebola viral diseases.

We also explored how the outbreaks of Ebola and Marburg impact the communities (Table 5). The community is usually engulfed with fear, especially the fear of deaths and the fear of the unknown cause of the disease. Initially, because of sudden deaths of many people especially in one family, they believe it's witchcraft. When Ebola virus is confirmed in their communities, business goes down drastically thus affecting them economically as was seen in West Africa [27–29]. The community also suffers from stigma from fellow citizens, but also if they go to hospitals, they may not receive treatment from the health workers. Other effects on the community include failure to participate in funeral rites of their loved ones, no social gatherings, and challenges associated with taking care of orphans and widows and widowers.

Regarding handling survivors of Ebola and Marburg virus disease, the communities do believe the person can recover completely from these diseases unless they have a letter from a health officer or the authority saying that the person has fully recovered. They reported that they would avoid the person for some time until they are sure the person is fully recovered.

On gender perspectives, they were contrasting views on whether it is men or women that are most affected by filovirus outbreaks. Although the majority believed that women were mostly affected, other participants said men are more affected by filovirus outbreaks. To sort out this conundrum, we used proportional piling that showed men scoring 55% compared to women with 45%. It shows that both men and women are almost equally affected during filovirus outbreaks (S5 Fig).

We explored ways on how best communication should best be done during outbreaks and community members ranked the community radio as the most effective means of communication followed by FM radio stations, village cooperative organizations and the community leaders (Table 5).

## Discussion

We found that EVD/MVD affected communities in Uganda are knowledgeable about EVD/MVD at 48.5% and 60% have a positive attitude towards control and prevention of these diseases. This is slightly higher than what has been found in similar studies towards Ebola virus disease especially in West Africa [18, 20, 30–38]. This is partly because Uganda has had many outbreaks of Ebola and Marburg viral diseases, which has led to continuous sensitization of communities to these diseases, hence change in attitude, and more knowledge gained. However, the proportion categorized as knowledgeable about EVD/MVD is still below average at 48%, and more sensitization is needed if future outbreaks are to be controlled in the shortest time possible. Community support and involvement are very key in control and prevention of VHFs given that this survey was done in communities that had exposure to VHF outbreaks, the knowledge levels could even be lower in other naïve communities. There is still a big proportion (above 51.5%) that is still less knowledgeable and have negative attitudes (40%) towards control and prevention, and these results should not be over-interpreted. One would have expected higher levels of knowledge given that the studied communities have experienced filovirus outbreaks twice.

Every outbreak that occurs is an opportunity to educate communities about a given disease, and Uganda has had EVD outbreaks five times [4–7] and MVD three times [8, 10, 39]. This should have provided the Ministry of Health of Uganda and other partners an opportunity to educate these communities on the modes of transmission, clinical symptoms, putative reservoirs and control and prevention methods. Although the communities demonstrate much fear towards Ebola and Marburg viruses, this can be advantageous for control and prevention measures as communities will be motivated to action if an outbreak occurs. However, this fear becomes counterproductive as far as survivors are concerned. Disease stigma is still an issue as 53% of the respondents said they would not associate with a survivor for fear of contracting the disease. Respondents reported that they would only associate with the survivor of EVD or MVD after careful evaluation and receiving a report from a health worker or the authority concerned. This was also observed in the 2001 EVD outbreak in Northern Uganda, as communities initially had their reservations about Ebola virus disease and survivors. However, after they had been explained to fully by the health care workers, survivors were accepted and are now living peacefully in their communities [40]. It is still hard for communities to fully accept that people completely recover from Ebola virus and that they can easily mix and interact with the rest of the community as evidenced in this research and from the West Africa EVD outbreak experience [20, 21].

Most participants mentioned that filoviruses spread fast, meaning they are highly contagious as was discussed in focused group discussions. Several modes of transmission were reported by the participants, which include contact with infected patients and contact or eating non-human primates and bats. This knowledge by the community is helpful during outbreaks in instituting control and prevention measures by health authorities. In communities that do not know modes of transmission, it would be difficult to stop the spread of the epidemic as was seen in West Africa [41]. However, we still have a few people who think that EVD and MVD are airborne, caused by witchcraft or by malice by medical workers from foreign countries. This was also revealed by de Vries *et al*. (2016) in an anthropological study in one of our study areas in Luweero district[22]. Such misconceptions need to be addressed because if taken on by opinion and community leaders as it happened in 2012 Luweero EVD outbreak, they could hamper prevention and control measures.

Participants highlighted bleeding symptoms as the most common sign of Ebola and Marburg viral disease (54%), and less than 20% indicated fever, diarrhea, and vomiting as a clinical sign of filoviruses. However, bleeding is not always there in all filovirus infected cases and usually comes at the end of the clinical course of the diseases [42]. It is important that both the public and clinicians know that hemorrhagic symptoms come later when the disease has progressed, and people who show hemorrhagic symptoms rarely recover. Early symptoms of filovirus infection are like those of any other infectious disease in the tropics, and they could easily be mistaken for malaria or typhoid. Hence mechanisms for early detection should be instituted to avoid missing cases as communities and clinicians wait to see hemorrhagic signs.

Sensitization of communities about filoviruses was the most effective means of control and prevention, as suggested by participants. They believe that if they are imparted with knowledge on the modes of transmission, control and prevention measures, and spread of the epidemic could be stopped in case there is an outbreak. They seemed not to understand though, the reasons why they could not participate in the long-held culture of funeral rites when they lose their loved ones. There is an information gap between health care providers and the affected communities on how filoviruses are transmitted and the how they should be managed. Although the community proposes other control measures such as quarantine and isolation of sick people and avoiding contact with infected patients, the feeling is that if they were fully sensitized about these methods before, during and after outbreaks would significantly reduce transmission chains during epidemics. Unlike the survey that was done in West Africa where TV was the most common source of information [20], participants in this study preferred the use of community radios as the most efficient way of passing on communication to the communities (Table 5). This model involves the use of loudspeakers placed in community trading centers where announcements can be made. Other preferred modes of communication during outbreaks included use include of FM local radio stations, use of village health teams and community leaders. These community-based strategies could prove to be efficient in communicating filovirus outbreaks instead of putting communication on TVs and other radio stations that do not broadcast in local languages and are city-based.

As seen in West Africa EVD outbreak 2014, filovirus outbreaks can be devastating since they change from localized disease outbreaks into a humanitarian crisis [43]. In this study, we see participants reporting several effects, which include the community being engulfed with fear of death or what they described as “the fear of unknown.” This fear of the unknown can lead to irrational decisions which can even potentiate the spread of the disease. This fear needs to be addressed early when the outbreak is detected and has been a missing link in many outbreaks of filoviruses. Communities tend to be isolated, and their business goes down drastically as other people from the same country do not want to associate with them. Another big complaint that is social-cultural in nature was a failure by the community to bury their loved ones. This needs to be addressed during outbreaks so that communities can feel like their loved ones have been buried in a proper way. Some African cultures believe that if someone is not buried in a proper manner, he will come back in real life to haunt the living family members.

Knowledge levels about EVD and MVD were different across different socio-demographic and other study variables. Being educated beyond primary level was the most significant predictor of awareness towards filoviruses. For example, people who have attained the secondary level of education were more likely to be knowledgeable about filoviruses as opposed to those who did not attain any formal education (OR = 3.6; 2.1–6.1). These odds were even higher for individuals who attained education beyond secondary school. This is correct because education is a key determinant of knowledge especially concerning health and health seeking behaviors and it has been found to influence people’s knowledge about EVD in Nigeria [18]. Education was still significant even after controlling for other variables such as sex and age. Males were more likely to be knowledgeable about filoviruses than females, possibly related to education because in many African societies, men are more apt to be more educated than women. However, males were still significant even after controlling for formal education meaning there are other contributing intrinsic factors. For example, information access may be more to men than women. People from MVD affected communities were more knowledgeable than people from EVD affected communities. Although the two communities come from two different tribes and live distant from each other one in the Western Uganda and another in Central Uganda, we do not find any plausible explanation as to why there should be a difference in the level of knowledge. This may, however, be influenced by the impact of the two diseases EVD being more pathogenic, causes more socio-cultural disruption leading to myths and misconceptions hence negative attitude and less knowledge about it. These factors were different from those reported by Iliyasu *et al*. (2015) [18] where the predictors of knowledge about EVD were being a health worker, being afraid about Ebola, and willingness to modify behavior. However, in another study in Nigeria, it was reported that education was a critical predictor of knowledge [44, 45], also indicated by comparing literacy rates of Uganda and West African countries. The countries that were affected by Ebola in West Africa have lower literacy rates compared to Uganda [46]. This could partly explain why people could not comprehend EVD as a disease in West Africa hence the increased transmission as compared to Uganda where people are more educated and experience low transmission rates of EVD to the extent of getting only one case in 2011 in Luweero district.

We explored gender disparities in this study using a proportional piling technique. Although many studies show that VHFs tend to affect more females than males because of their gender roles [47, 48], our study revealed that almost all men and women are affected equally (S5 Fig). However, from the FGDs, men were indicated as more likely to be index cases than women because of their risky behavior and gender roles such as hunting, clearing land for agriculture and going into the forest for several activities. As the outbreak progresses, women tend to be more likely to be affected since they are more into caring for the sick hence have higher chances of being infected.

These results from our study may not be generalized to the communities in the whole of Uganda. Studied communities were selected purposively because of their previous experience with Ebola and Marburg outbreaks. Communities that have experienced outbreaks are more likely to have received education through social mobilizations that happened during outbreaks, and hence appear to be more knowledgeable than other communities that have not experienced outbreaks. Another limitation of this study could be possibly biased responses drawing from outbreak experiences. Probably the answers and knowledge assessment outcomes would be different if the same study is done in an entirely naïve population.

### Conclusion

In conclusion, the study revealed that communities in Uganda that had been affected by filovirus outbreaks are slightly knowledgeable and have a good attitude towards control and prevention of EVD. Formal education is a significant predictor of knowledge and attitude towards filoviruses. Communities could identify the suspect cases and are aware of the modes of transmission, and they suggest sensitization as the best approach for control of filovirus outbreaks. Although Uganda health sector has developed preparedness plans to respond to filovirus outbreaks, the level of knowledge about filoviruses is still below average and needs to be improved. The public health sector could enhance communities’ knowledge and attitude by supplying more educational materials and conducting health education for epidemic preparedness and using appropriate communication channels as proposed by the communities.

## Supporting information

S1 Questionnaire Questionnaire that was used to collect quantitative data. (PDF)

S1 FGD guide Focused group discussion guide that was used to collect qualitative data. (PDF)

S1 Fig This is a picture showing how participants in one of the FGDs ranked the most important clinical signs of Ebola Virus disease. The clinical signs are written in one of the local languages in Uganda, Luganda.
(TIF)

S2 Fig This picture shows proportional piling technique where participants used 100 grains of beans to distribute them according to what they think is most important in transmitting Ebola Virus disease. Words are written in the local language, Luganda.
(TIF)

S3 Fig The picture shows pairwise ranking technique where participants listed and compared the possible causes of filovirus outbreaks among themselves to come up with a rank of the most important cause. Causes were listed in both rows and columns in the local language.
(TIF)

S4 Fig The Receiver Operating Curve(ROC) that was used to assess the model for predictors of knowledge towards Ebola and Marburg virus diseases. (TIF)

S5 Fig This picture shows proportional piling of 100 grains of beans to determine which gender is affected most by filovirus outbreaks. (TIF)

S1 Table Themes and categories generated from focused group discussions by conventional content analysis technique about People’s knowledge and attitude towards Ebola and Marburg virus diseases. (DOCX)

S2 Table Results of pairwise ranking technique applied on risk factors/causes of Ebola and Marburg virus diseases. (DOCX)

S3 Table An alternative model to the logistic regression model if no categorisation of knowledge is done. The predictors of knowledge score are the same as those in the logistic regression.
(XLSX)

S1 Data Quantitative data set. (XLSX)
