# Supplementary material for: Knowledge and attitude towards Ebola and Marburg virus diseases in Uganda using quantitative and participatory epidemiology techniques
Source: PLoS Negl Trop Dis. 2017 Sep 11;11(9):e0005907. doi: 10.1371/journal.pntd.0005907 (PMC5608436; doi:10.1371/journal.pntd.0005907)
Supplement: S1 Questionnaire — (PDF) [file pntd.0005907.s001.pdf]

Questionnaire

Date: ____/____/_____ (D, M, Yr) **Sample ID**_________________________**Participant classification**___________________

Form Completed by:

Name: ______________________ Position: ________________District: ______________________

Phone Number/email: ____

Section 1. Assessment Participant Information

ID Number: ____________________ Name_______________________________________Age: _________Gender: ☐ Male ☐ Female.

Village/Town: ________________Parish: _____________Sub-County: _________________

District: _________________Nationality: _____________ Marital status ☐ Married ☐ Single ☐ Widowed

GPS Coordinates____

1. **Education level** ☐ None ☐ Primary ☐ Secondary ☐ Post-Secondary ☐ Other specify_____
2. **Primary Occupation:** ☐ Farmer ☐ Herdsman ☐ Housewife ☐ Student ☐ Child ☐ Animal Health worker ☐ butcher ☐ Trader ☐ Other, please specify occupation____

Knowledge & Attitude Questions

3. Have you heard about Ebola or Marburg Virus Diseases ☐ Yes ☐ No ☐ Unk
4. If yes, from whom: ☐ Health worker ☐ Radio ☐ community leaders ☐ others specify________
5. Do you know the signs and symptoms of Ebola or Marburg virus diseases in humans ☐ Yes ☐ No ☐ Unk
6. If yes how ☐bleeding ☐ High fever ☐ vomiting ☐ diarrhea ☐others specify_____________
7. Do you know the signs and symptoms of Ebola or Marburg virus diseases in animals ☐ Yes ☐ No ☐ Unk
8. If yes how ☐bleeding ☐ High fever ☐ nasal discharge ☐ diarrhea ☐ abortion ☐ reduced milk production ☐others specify_____________
9. Do you know who to contact in case you see a suspect case of Ebola or Marburg diseases? ☐ Yes ☐ No ☐ Unk
10. Do you believe Ebola or Marburg diseases really exists ☐ Yes ☐ No ☐ Unk If no, why____
11. Have you heard of any survivor of Ebola or Marburg diseases ☐ Yes ☐ No ☐ Unk
12. Would you relate/interact with a survivor of Ebola or Marburg diseases ☐ Yes ☐ No ☐ Unk
13. Would you welcome someone back into their community/neighborhood after a neighbor has recovered from Ebola or Marburg diseases ☐ Yes ☐ No ☐ Unk
14. If no, why ☐ fear of contracting disease ☐ fear of stigma from community ☐ others specify____________
15. Do you know how Ebola or Marburg disease is transmitted ☐ Yes ☐ No ☐ Unk
16. If yes, how ☐ body contact sick person ☐ through air ☐ through needle pricks ☐ contact with animals ☐contact with dead person ☐ contact with body fluids of sick person ☐biting mosquitoes(insects) ☐ others specify_________________
17. If transmission through animals, which ones ☐ goats ☐ Cattle ☐ Sheep ☐ Poultry ☐ Dogs ☐ monkeys ☐ bats ☐ antelopes ☐ wild pigs ☐ others specify______________
18. How do you think you can protect yourself from acquiring Ebola or Marburg disease? ☐ vaccination ☐ avoiding contact with animals ☐ traditional medicine ☐ avoiding sick people ☐ sleeping in a mosquito net ☐others specify____
19. How do you think Ebola or Marburg diseases can best be healed or treated? ☐ traditional medicine ☐ spiritual healing ☐ Modern medicine ☐Herbal medicine ☐others specify_________________
20. Do you think you are at risk of contracting Ebola or Marburg virus disease ☐ Yes ☐ No ☐ Unk
21. If yes/no, why____

22. Do you know how Ebola or Marburg diseases can be prevented ☐ Yes ☐ No ☐ Unk
23. If yes how____

Thank you for your Time

End of Interview
