# Supplementary material for: Knowledge and attitude towards Ebola and Marburg virus diseases in Uganda using quantitative and participatory epidemiology techniques
Source: PLoS Negl Trop Dis. 2017 Sep 11;11(9):e0005907. doi: 10.1371/journal.pntd.0005907 (PMC5608436; doi:10.1371/journal.pntd.0005907)
Supplement: S1 FGD guide — (PDF) [file pntd.0005907.s002.pdf]

**Guide for Focus Group Discussions**

1. What do you think about Ebola?

   (Probe) Do you know how to identify a suspect of Ebola?
2. What causes Ebola outbreaks?
3. How do you think Ebola is transmitted?
4. What knowledge do you possess in managing Ebola?

   (Probe) How would you prevent yourself from acquiring Ebola?
5. Do the health facilities in your community capable of managing Ebola?
6. What was your experience during the EVD outbreak?
7. Can you associate with an Ebola survivor?
8. Does Ebola affect men and women differently?
9. What would be the best way of communicating an Ebola outbreak in your community?

   (Probe) How can epidemic awareness be improved in your community?
10. How can people’s perception of Ebola be improved?

    (Probe) Does Ebola have an age group it mostly affects?
