# Supplementary figures and images for: Knowledge and attitude towards Ebola and Marburg virus diseases in Uganda using quantitative and participatory epidemiology techniques
Source: PLoS Negl Trop Dis. 2017 Sep 11;11(9):e0005907. doi: 10.1371/journal.pntd.0005907 (PMC5608436; doi:10.1371/journal.pntd.0005907)

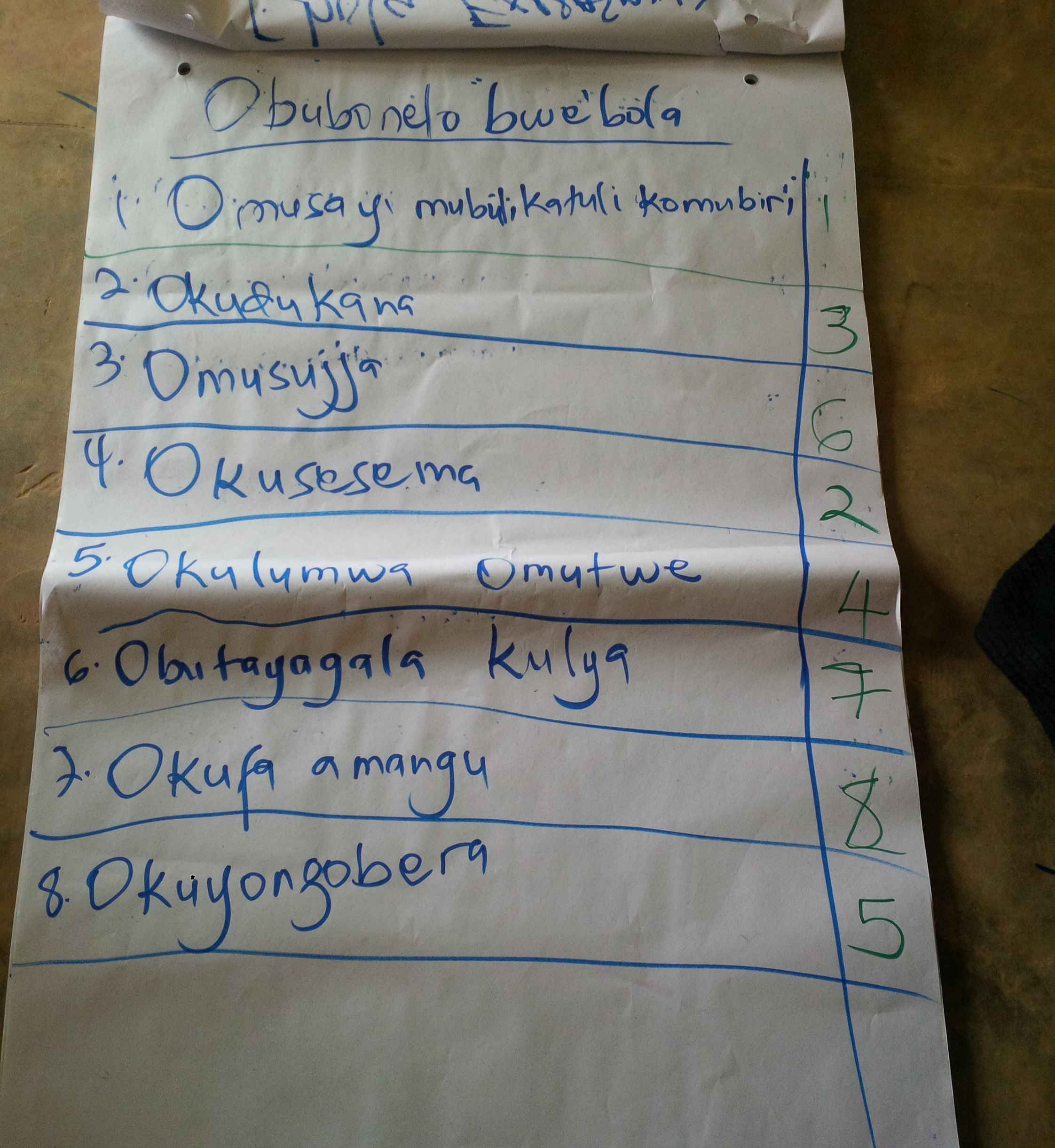

Supplement: S1 Fig — The clinical signs are written in one of the local languages in Uganda, Luganda. (TIF) [file pntd.0005907.s003.tif]

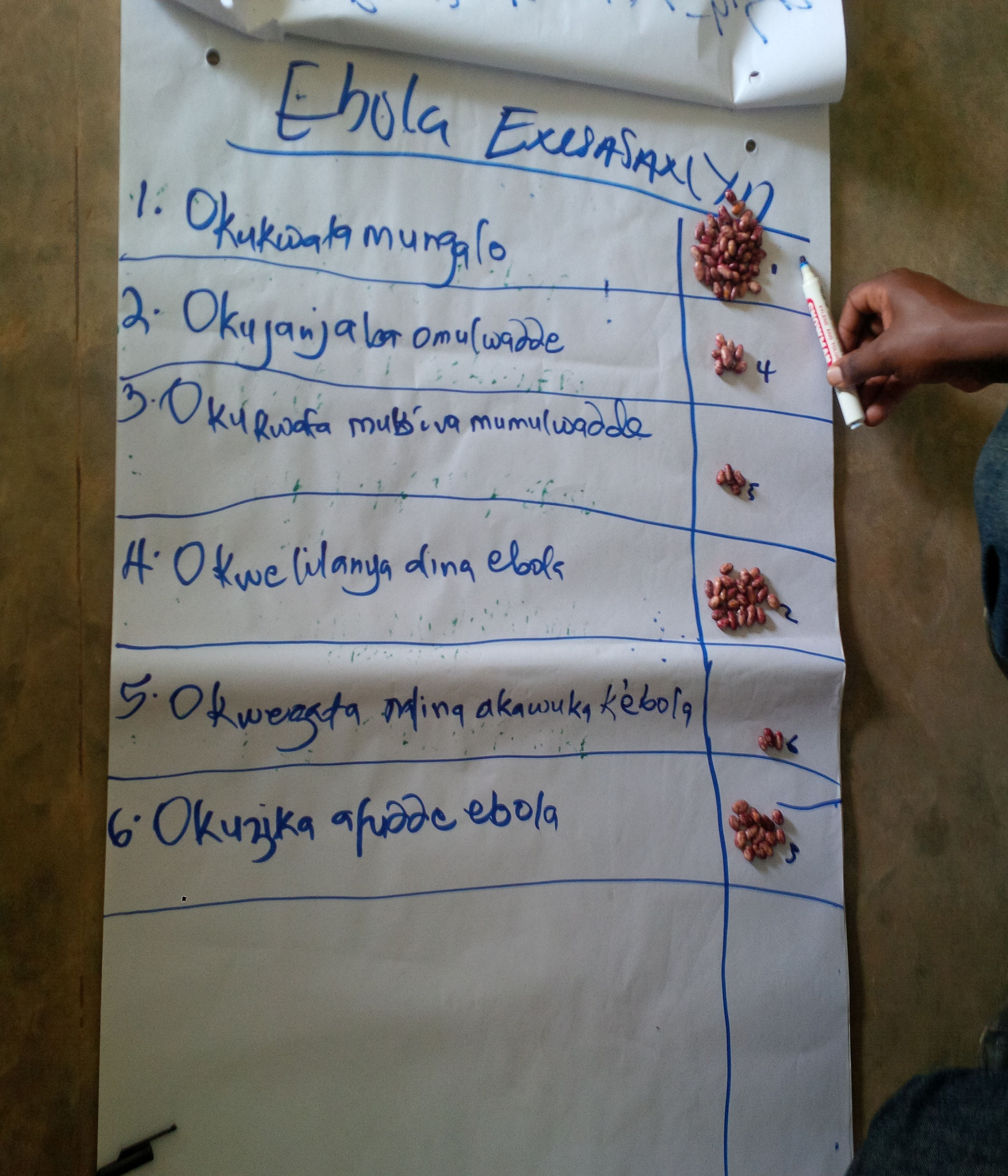

Supplement: S2 Fig — Words are written in the local language, Luganda. (TIF) [file pntd.0005907.s004.tif]

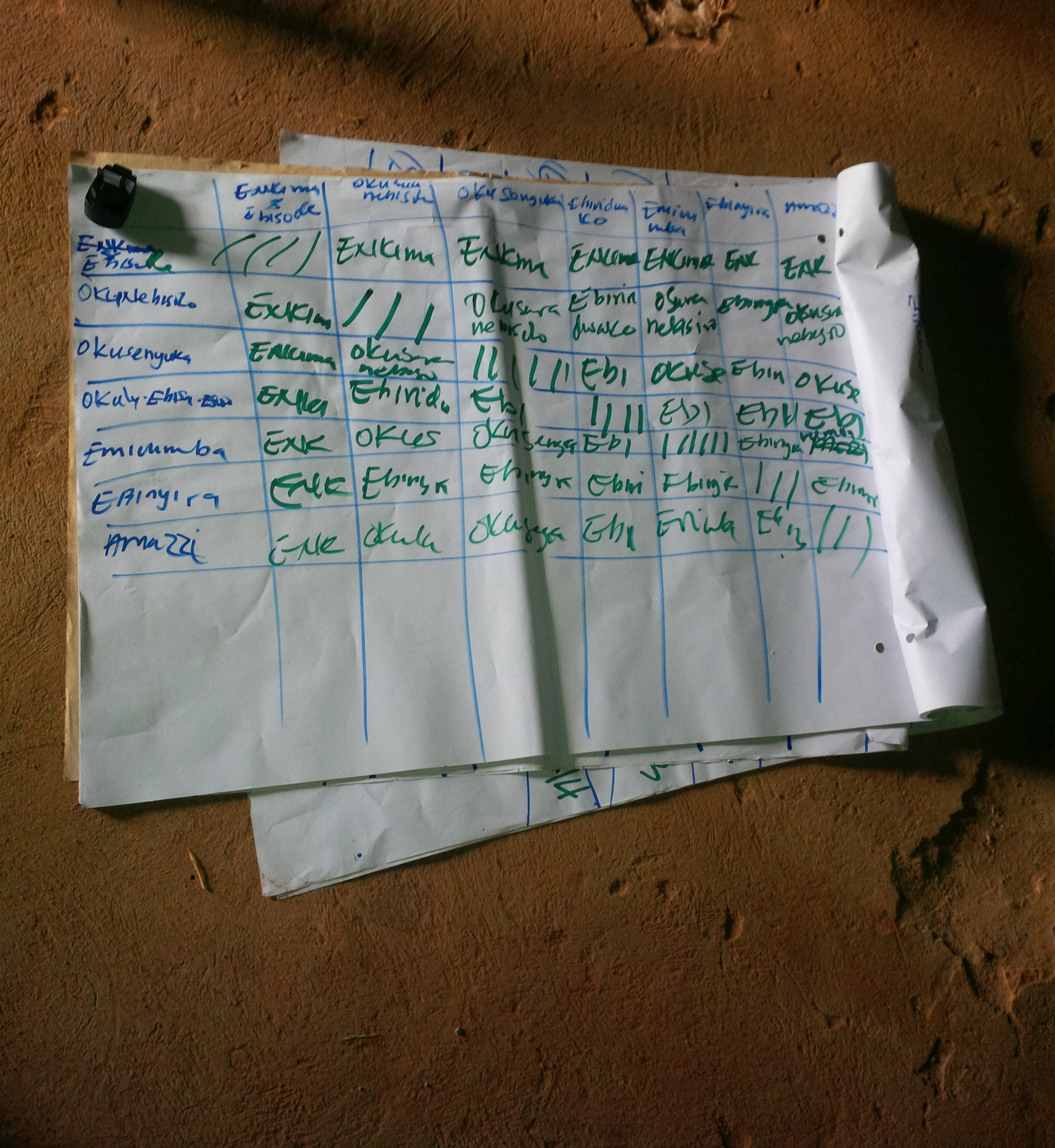

Supplement: S3 Fig — Causes were listed in both rows and columns in the local language. (TIF) [file pntd.0005907.s005.tif]

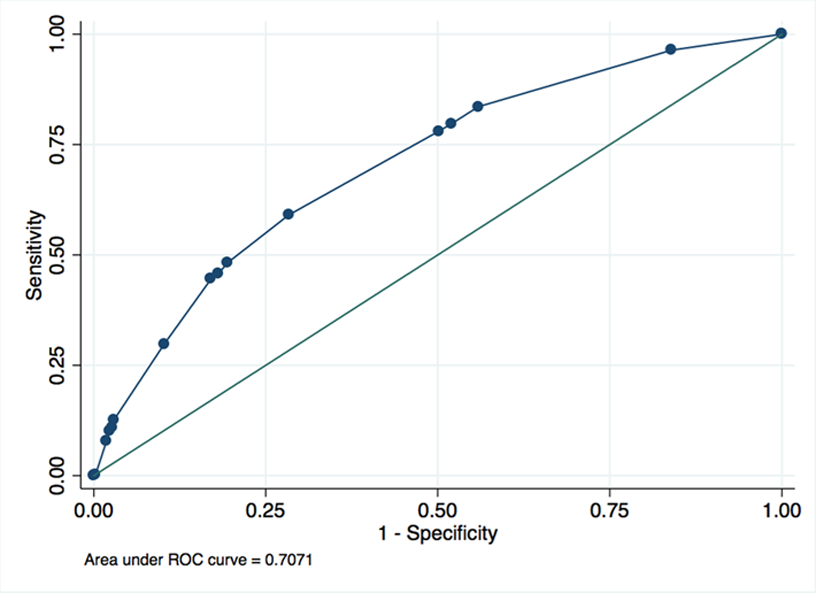

Supplement: S4 Fig — (TIF) [file pntd.0005907.s006.tif]

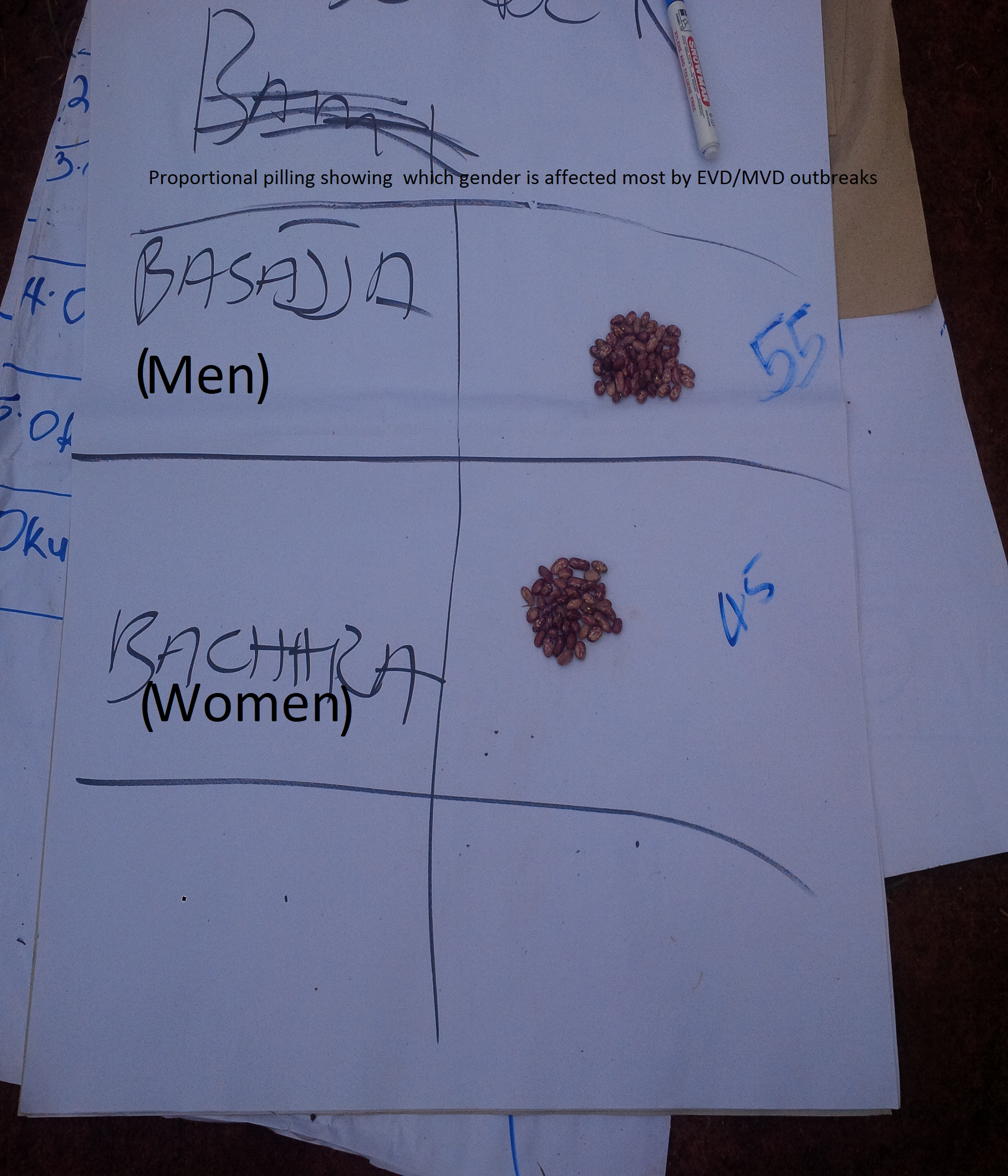

Supplement: S5 Fig — (TIF) [file pntd.0005907.s007.tif]
