# Supplementary material for: Knowledge and attitude towards Ebola and Marburg virus diseases in Uganda using quantitative and participatory epidemiology techniques
Source: PLoS Negl Trop Dis. 2017 Sep 11;11(9):e0005907. doi: 10.1371/journal.pntd.0005907 (PMC5608436; doi:10.1371/journal.pntd.0005907)
Supplement: S1 Table — (DOCX) [file pntd.0005907.s008.docx]

**Themes and Categories generated from Focused Group Discussions by Conventional Content Analysis technique about People’s knowledge and Attitude towards Ebola Virus Diseases and Marburg Virus Disease in Uganda.**

| Category | Themes | Key quotes |
| --- | --- | --- |
| ‘Fear’ (Ebola Kills instantly) | More pathogenic than AIDS  Causes chaos  It is very small-microscopic  Terrible disease  Kills fast, severe disease | “When I hear Ebola, I lose strength because it kills instantly” (FGD 1)  “When you get Ebola, your life ends there” (FGD2) |
| Cause and mode of spread  “Spreads very fast.” | No funeral rites  Contact (Touching people with Ebola or Marburg, you get infected, other means of contact with dead or alive infected person  Ebola disease is airborne  Animals cause the disease-monkeys (Eating fruits eaten on by monkeys, through wild animals such as bats, sharing shelter with animals, eating monkeys)  Migration of people from affected countries like South Sudan and DRC, wearing second-hand clothes, sharing sharp instruments with patients  Hygiene is key- Ebola is caused by poor hygiene  Caused by malice and bioterrorism by doctors | “How come in Uganda Ebola kills only one person because it is known to kill many people” (FGD4)  “*Omulya mamba era omu, navumaganya ekikka*” meaning when ‘one member of a clan eats a totem, he brings shame to whole clan’ referring to those people who eat wild animals like monkeys. (FGD4)  Maybe some doctors make the virus and use it to make money (FGD5)  “*Abazungu balesse obulwadde bakendenze ku namba yabantu*” meaning ‘foreigners have brought the disease to reduce the population of the world’ (FGD4) |
| Clinical presentation | Vomiting, diarrhea, red eyes, high fever, oozing of blood from body orifices, body weakness, headache, abdominal pains, sweating | “I have never seen such a deadly disease since my daughter started falling sick with a simple fever and we all thought it was malaria” (FGD3) |
| Control and prevention | Sensitization during and after the outbreak is crucial for outbreak control  Safe burial (have designated Ebola burial sites, provide masks to people who are going to bury, train local teams to bury their people)  Quarantine or isolate sick people  Treatment and recruitment of Ebola trained health workers and Reporting to health workers  Avoid contact (sick people, animals  Kill rats and bats | “*Adingana amawolu yagajamu omukuto*” meaning repetitive sensitization should be done before Ebola outbreaks (FGD1) |
| Community Response & Impact | Initially thought it was witchcraft (because people die in the same family), don’t believe that its Ebola or Marburg diseases  Business is at standstill  Pressure, worry, and Fear of the unknown  Failure to practice funeral rites  Death of loved ones  Stigma, orphans, and survivors neglected and stigmatized by others  Improper burial has spiritual impact | “People think that Ebola is brought by doctors from outside countries” (FGD5)  “People call me Ebola now, and they think I got much money from the government” (FGD5) |
| Stigma and Fear of survivors | Avoid the survivor for some time; You can't be sure that he has completely healed, some people can escape from isolation units  Need to consult the doctors before someone can be accepted  Survivor should come with Authority or doctor to community  Affected communities get stigmatized by fellow countrymen, their products are not bought, and there is total breakdown of business  Neglected by health workers | “Pictures of people infected and survivors should be published so that they can be avoided” (FGD1)  “If you see an Ebola Patient, Run for your life” (FGD1)  “I try to get written proof that the person is ok.” |
| Gender perspectives | Women were most affected because they are the caretakers  Causes separation in homes  Kills men who are breadwinners  Affects men mostly, since women have monthly menstrual periods, hence releasing the disease, also its men that look for trouble  Affects both men and women equally | “Since men travel a lot, they should be the transmitters of Ebola” (FGD 5) |
| Communication during outbreaks | Radios, Use of Village Health Teams (VHTs) and Television  House to house, posters, and pictures, mobile Vans, Mobile phones, worship areas, use of local leaders, Music dance and drama  Community leaders and radios, new papers |  |
