# Supplementary material for: Knowledge and attitude towards Ebola and Marburg virus diseases in Uganda using quantitative and participatory epidemiology techniques
Source: PLoS Negl Trop Dis. 2017 Sep 11;11(9):e0005907. doi: 10.1371/journal.pntd.0005907 (PMC5608436; doi:10.1371/journal.pntd.0005907)
Supplement: S2 Table — (DOCX) [file pntd.0005907.s009.docx]

**Results of Pairwise Ranking technique applied on risk factors/causes of Ebola and Marburg virus diseases**

| Risk Factor | Primates | Sleeping in the same house with animals | Migration of people | Eating fruits eaten on by animals | Second-hand clothes | Bats | Dirty Water | Score | Ranks |
| --- | --- | --- | --- | --- | --- | --- | --- | --- | --- |
| Primates |  | Primates | Primates | Primates | Primates | Primates | Primates | 12 | 1 |
| Sleeping in the same house with animals | Primates |  | Sleeping in the same house with animals | Eating fruits eaten on by animals | Sleeping in the same house with animals | Bats | Sleeping in the same house with animals | 6 | 4 |
| Migration of people | Primates | Sleeping in the same house with animals |  | Eating fruits eaten on by animals | Migration of people | Bats | Migration of people | 4 | 5 |
| Eating fruits eaten on by animals | Primates | Eating fruits eaten on by animals | Eating fruits eaten on by animals |  | Eating fruits eaten on by animals | Bats | Eating fruits eaten on by animals | 8 | 3 |
| Second-hand clothes | Primates | Sleeping in the same house with animals | Migration of people | Eating fruits eaten on by animals |  | bats | Second-hand clothes | 2 | 6 |
| Bats | Primates | Bats | Bats | Bats | Bats |  | Bats | 10 | 2 |
| Dirty water | Primates | sleeping in the same house with animals | Migration of people | Eating fruits eaten on by animals | Second-hand clothes | bats |  | 0 | 7 |
